# Community carriage of methicillin-resistant staphylococci among migrant communities living in Klang Valley, Malaysia

**DOI:** 10.1016/j.ijregi.2026.100849

**Published:** 2026-01-24

**Authors:** Nurul Amirah Mohamad Farook, Adrian Anthony Pereira, Thana Seelan, Sabrina Di Gregorio, Nor Azila Muhammad Azami, Hui-min Neoh

**Affiliations:** 1 UKM Medical Molecular Biology Institute (UMBI), Universiti Kebangsaan Malaysia, Kuala Lumpur, Malaysia; 2 North South Initiative (NSI), Petaling Jaya, Selangor, Malaysia; 3 Universidad de Buenos Aires, Facultad de Farmacia y Bioquímica, Instituto de Bacteriología y Virología Molecular, Buenos Aires, Argentina; 4 CONICET, Buenos Aires, Argentina; 5 UKM Pakarunding Sdn. Bhd. (UKMPSB), Selangor, Malaysia

**Keywords:** Methicillin-resistant staphylococci, MRCoNS, MRSA, *S. epidermidis*, *S. haemolyticus*, Pink-collar industry

## Abstract

**Objectives:**

This study aimed to determine community carriage of methicillin-resistant *Staphylococcus aureus* (MRSA), *S. epidermidis* (MRSE), and *S. haemolyticus* (MRSH) among migrant workers from Indonesia, Bangladesh, and Nepal residing in Klang Valley, Malaysia*.*

**Methods:**

A total of 258 migrant workers were recruited for nasal swab screening of MRSA, MRSH and MRSE. Swabs were cultured on CHROMagar™ MRSA, and colonies exhibiting growth were subjected to species identification, antibiotic susceptibility testing and SCC*mec* typing.

**Results:**

Among 258 participants, 38 (14.7%) carried methicillin-resistant staphylococci (MRS), with Indonesian workers having the highest prevalence (n = 19, 50.0%). Among the MRS, MRSH was mostly commonly isolated (n = 18, 40.0%), followed by MRSA (n = 11, 24.4%) and MRSE (n = 2, 4.4%). Many isolates were resistant to erythromycin (n = 24, 53.3%), tetracycline (n = 17, 37.8%), ciprofloxacin (n = 17, 37.8%) and clindamycin (n = 17, 37.8%). SCC*mec* types I and V were predominantly found in MRSH (n = 20, 80.0%), while type IV was most common in MRSA (n = 10, 90.9%) and MRSE (n = 5, 55.6%).

**Conclusions:**

This is the first report of community carriage MRS in migrant workers living in Malaysia. Silent MRS carriage in pink-collar industries, such as domestic helpers might have local community transmission implications and could be further investigated.

## Highlights

• Migrant workers in Malaysia carry methicillin-resistant staphylococci (MRS).
• Crowded living conditions are significantly linked to migrant MRS community carriage.
• Varied antibiotic resistance profiles in migrant community carriage MRS.
• MRS carriage among pink-collar migrant workers warrants surveillance.

## Introduction

Economic migrants cross international borders to seek better employment opportunities in foreign countries. Many of these communities make important contributions to their host countries; however, at the same time, migrants might not receive equal health care coverage that local citizens do. Most economic migrants are usually housed in dormitories or shared accommodations, which increases the risk of disease transmission.

Methicillin-resistant *Staphylococcus aureus* (MRSA) is a significant pathogen associated with both hospital- and community-acquired infections. Similar with other staphylococci, it can be silently carried in the anterior nares of healthy individuals, and poses risks for subsequent infections and transmission. While MRSA has been extensively studied, investigations on methicillin-resistant coagulase-negative staphylococci (MRCoNS) such as *Staphylococcus epidermidis* (MRSE) and *Staphylococcus haemolyticus* (MRSH) remain few despite their increasing clinical significance [1]. In Malaysia, carriage of these bacteria within the community setting remains largely unknown, particularly among migrant workers, who may act as hidden reservoirs for silent transmission.

In this study, we conducted a preliminary screening to assess the silent carriage of methicillin-resistant staphylococci (MRS) among migrant workers originating from Indonesia, Bangladesh, and Nepal living in Klang Valley, a major economic hub in Malaysia. Antibiotic susceptibility testing and Staphylococcal cassette chromosome *mec* (SCC*mec*) genotyping of isolated MRS were then performed.

## Methods

### Participant recruitment

Migrant workers originating from Indonesia, Bangladesh, and Nepal who were living in Klang Valley were recruited through the North-South Initiative (NSI), a Malaysian non-governmental organization between December 2023 and May 2024. Participants (aged ≥18 years) were briefed on the study before consent-taking and sociodemographic data collection (Supplementary Methods). Sample collection was conducted at the NSI headquarters, where one nasal swab (both anterior nares) was obtained from each participant. Swabs were transported in modified Amies transport medium (Vacutest Kima, Italy) at room temperature and processed within 24 hours.

### MRS isolation and species identification

Collected swabs were directly inoculated on CHROMagar™ MRSA (Paris, France) agar. Grown colonies were morphologically examined (MRSA; mauve colonies and MRCoNS; white colonies). Genomic DNA of isolated MRS strains were then extracted via lysostaphin digestion; MRS was confirmed using polymerase chain reaction (PCR) amplification with species-specific primers and detection of *mec*A gene (Supplementary Methods).

### Antibiotic susceptibility testing and SCCmec typing

Strains were tested for their antibiotic susceptibilities using the AST-GP67 card in a VITEK-2 automated system (bioMérieux). SCC*mec* typing was performed using multiplex PCR as described previously to further characterize the confirmed MRS strains (Supplementary Methods). Multiplex PCR was performed with the following conditions; one cycle of initial denaturation at 94°C for 4 minutes, 30 cycles of denaturation at 94°C for 30 seconds, annealing for 30 seconds at 55°C, extension at 72°C for 1 minute, and one cycle of final extension at 72°C for 4 minutes.

### Statistical analysis

All statistical analyses and data visualizations were performed using R version 4.4.2. Categorical variables were summarized as frequencies, and association of MRS carriage were compared using chi-square or Fisher’s exact tests, where *P*-value of <0.05 was considered as statistically significant.

## Results

We successfully recruited 258 participants for the study (Indonesians, n = 159, Bangladeshis, n = 51 and Nepalis, n = 48). Among the participants, 38 (14.7%) were MRS-positive and their distribution is summarized in Table 1. Most were from Indonesia (n = 19, 50%), followed by Bangladesh (n = 15, 39.5%) and Nepal (n = 4, 10.5%). Seven out of 38 participants (18.4%) carried both MRSH and MRSE, but none co-carried MRSA. MRS carriage was significantly associated with the participant’s nationality (*P* = 0.004) and the MRS species detected (*P* < 0.001). Investigation into sociodemographic factors, including gender, work industry, antibiotic intake, hospitalization and living arrangements found no significant association with MRS carriage except for number of roommates (*P* = 0.014). Most of the participants carrying MRS lived in shared accommodation (n = 23, 60.5%), with an average of seven individuals per household, demonstrating the association between MRS carriage and crowded living conditions.

**Table 1:** Distribution of migrant participants (n = 258) and MRS carriers (n = 38) included in this study.

Study characteristics	n (%)					*P*-value^a^
	Migrant participant	MRS carriers	MRS carriers categorized based on country			
			Indonesia	Bangladesh	Nepal	
**Total**	258	38	19	15	4	
**Gender**						0.824
Male	137 (53.1)	21 (55.2)	4 (21.1)	14 (93.3)	3 (75.0)	
Female	116 (44.9)	15 (39.5)	14 (73.7)	1 (6.7)	0 (0.0)	
Not available	5 (2.0)	2 (5.3)	1 (5.3)	0 (0.0)	1 (25.0)	
**Industry**						0.431
Domestic worker	133 (51.6)	15 (39.5)	15 (78.9)	0 (0.0)	0 (0.0)	
Manufacturing	46 (17.8)	10 (26.3)	0 (0.0)	10 (66.7)	0 (0.0)	
Construction	24 (9.3)	6 (15.8)	1 (5.3)	5 (33.3)	0 (0.0)	
Food and beverage	14 (5.4)	4 (10.5)	3 (15.8)	0 (0.0)	1 (25.0)	
Security	6 (2.3)	2 (5.3)	0 (0.0)	0 (0.0)	2 (50.0)	
Retail and services	4 (1.6)	1 (2.6)	0 (0.0)	0 (0.0)	1 (25.0)	
Others	14 (5.4)	0 (0.0)	0 (0.0)	0 (0.0)	0 (0.0)	
Not available	17 (6.6)	0 (0.0)	0 (0.0)	0 (0.0)	0 (0.0)	
**Living arrangement**						1
Shared accommodation	199 (77.1)	30 (78.9)	15 (78.9)	14 (93.3)	1 (25.0)	
Alone	57 (22.1)	8 (21.1)	4 (21.1)	1 (6.7)	3 (75.0)	
Not available	2 (0.8)	0 (0.0)	0 (0.0)	0 (0.0)	0 (0.0)	
**Number of roommates (if shared accommodation)**						**0.014**
1-5 roommates	88 (34.1)	16 (42.1)	7 (36.8)	9 (60.0)	0 (0.0)	
6-10 roommates	54 (20.9)	7 (18.4)	6 (31.6)	1 (6.7)	0 (0.0)	
>10 roommates	6 (2.3)	4 (10.5)	0 (0.0)	4 (26.7)	0 (0.0)	
Not available	51 (19.8)	3 (7.9)	2 (10.5)	0 (0.0)	1 (25.0)	
**History of hospitalization (last 6 months)**						1
Yes	12 (4.6)	2 (5.3)	1 (5.3)	1 (6.7)	0 (0.0)	
No	244 (94.6)	36 (94.7)	18 (94.7)	14 (93.3)	4 (100.0)	
Not available	2 (0.8)	0 (0.0)	0 (0.0)	0 (0.0)	0 (0.0)	
**History of antibiotics intake (last 6 months)**						0.077
Yes	41 (15.9)	9 (23.7)	4 (21.1)	3 (20.0)	1 (25.0)	
No	202 (78.3)	25 (65.8)	12 (63.2)	12 (80.0)	3 (75.0)	
Not sure	9 (3.5)	3 (7.9)	3 (15.8)	0 (0.0)	0 (0.0)	
Not available	6 (2.3)	1 (2.6)	0 (0.0)	1 (6.7)	0 (0.0)	
**MRS carriage**						**0.001**
MRSA	11 (4.3)	11 (28.9)	1 (5.3)	10 (66.7)	0 (0.0)	
MRSH	18 (7.1)	18 (47.4)	11 (57.9)	5 (33.3)	2 (50.0)	
MRSE	2 (0.8)	2 (5.3)	2 (10.5)	0 (0.0)	0 (0.0)	
MRSH + MRSE	7 (2.8)	7 (18.4)	5 (26.3)	0 (0.0)	2 (50.0)	

MRS, methicillin-resistant staphylococci; MRSA, methicillin-resistant *Staphylococcus aureus*; MRSE, methicillin-resistant *S. epidermidis*; MRSH, methicillin-resistant *S. haemolyticus*.

a *P* <0.05 indicates statistically significant (bold).

**Figure 1:**
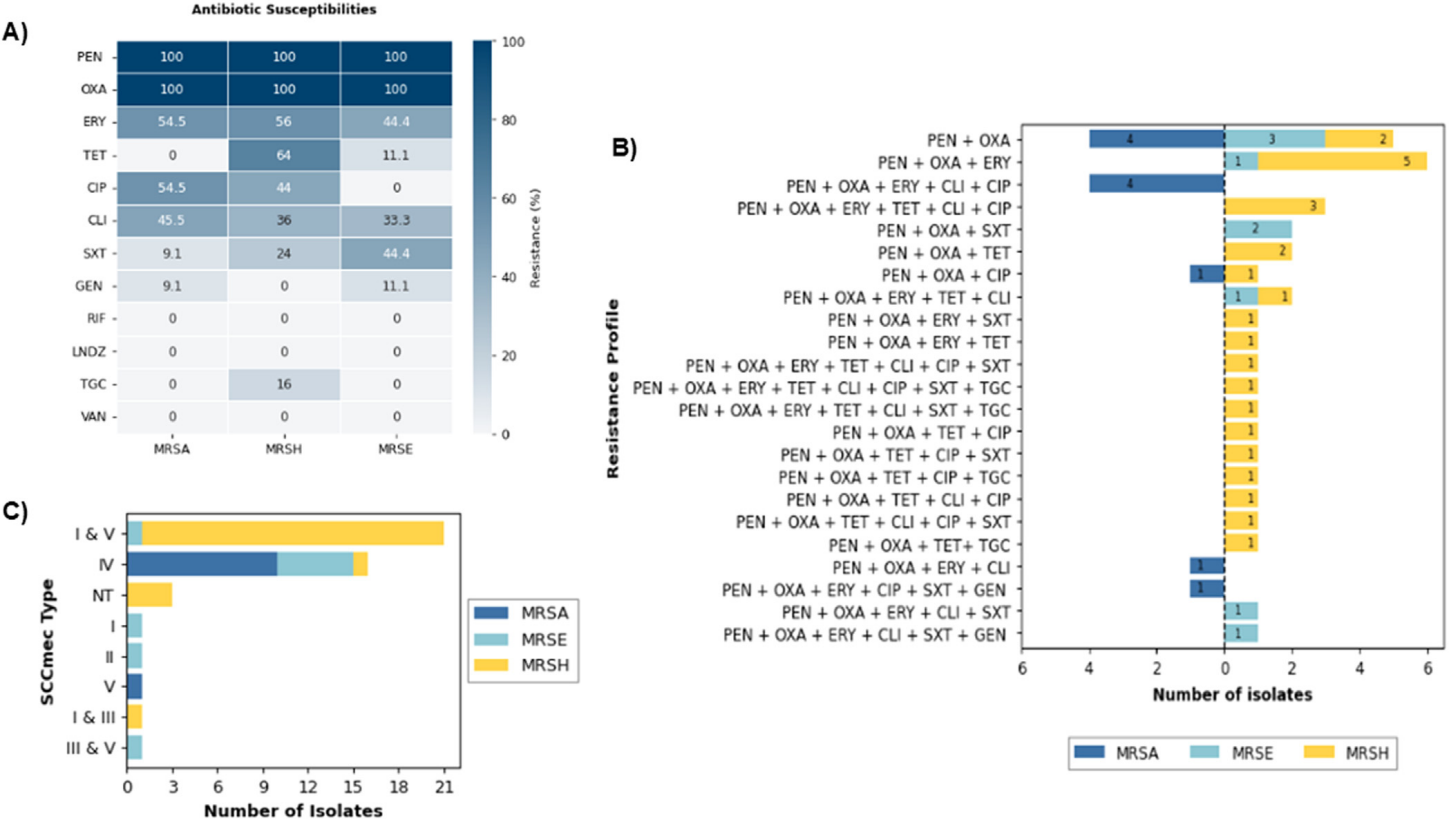
Phenotypic and molecular characteristics of MRSA, MRSH, and MRSE isolated in this study. (a) Antibiotic susceptibilities expressed as percentage of resistance (b) Resistance profiles (c) Distribution of SCC*mec* types. Abbreviations: CLI, clindamycin; CIP, ciprofloxacin; ERY, erythromycin; GEN, gentamicin; LNDZ, linezolid; NT, non-typeable; OXA, oxacillin; PEN, penicillin; RIF, rifampicin; SXT, trimethoprim/sulfamethoxazole; TGC, tigecycline; TET, tetracycline; VAN, vancomycin.

Overall, a total of 45 MRS was recovered, with MRSH more commonly isolated among the MRS (n = 18, 40.0%). MRSA and MRSE were detected in 11 (24.4%) and two (4.4%) from the total isolated MRS, respectively. All MRS were resistant to penicillin and oxacillin. More than half were additionally resistant to erythromycin (n = 24, 53.3%) followed by tetracycline (n = 17, 37.8%), ciprofloxacin (n = 17, 37.8%) and clindamycin (n = 17, 37.8%) (Figure 1a). Comparatively, fewer isolates were resistant against trimethoprim/sulfamethoxazole (n = 11, 24.4%), tigecycline (n = 4, 8.9%) and gentamicin (n = 2, 4.4%). More MRSA strains were found to be resistant to clindamycin and ciprofloxacin compared to the MRCoNS of this study (Figure 1a), although the latter exhibited broader resistance profiles compared to MRSA. SCC*mec* types I and V were the most common among our study isolates, and predominantly found in MRSH (n = 20, 80.0%). In contrast, most MRSA (n = 10, 90.9%) and MRSE (n = 5, 55.6%) isolates harbored SCC*mec* type IV (Figure 1c).

## Discussion and conclusion

Malaysia hosts over 2.2 million migrant workers from diverse regions; however, data on their carriage of antibiotic-resistant bacteria remains largely unknown. We hypothesized that MRS nasal carriage patterns and prevalence vary among migrant workers from different nationalities. Our findings revealed a 14.7% MRS carriage rate, with distinct species predominating in different nationalities. MRSH, the most isolated species, was common among Indonesian workers, many of whom were employed in pink-collar industries. In contrast, MRSA was more commonly found in Bangladeshi workers, while Nepali workers had relatively low carriage rates of either species. A study in Taiwan involving foreign workers reported a MRSA carriage rate of 2.72%, with the highest carriage among Vietnamese workers [2]. The higher rate observed in our study (4.3%, n = 11/258) may be attributed by the inclusion of Bangladeshi workers, who had higher MRSA carriage but were not screened in the Taiwanese cohort. Nonetheless, these rates may not fully represent the true prevalence due to selection bias of study participants.

Differences in MRS nasal carriage could be influenced by factors such as hygiene practices, healthcare access, and living conditions [3]. In our study, the number of roommates in migrant shared accommodation was significantly associated with MRS carriage. Overcrowded shared accommodations, with some having >10 roommates per house could have facilitated MRSA and MRSH transmission among the Indonesian and Bangladeshi workers. Such living conditions have been associated with increased MRSA transmission, due to possible close contact and contaminated surfaces; in contrast to Nepali workers who mostly lived alone and had less exposure to these risk factors [4]. Additionally, although previous antibiotic intake and hospital admission often influenced MRS nasal carriage and host colonization [5], no such associations were observed in this study. Intriguingly, none of the individuals colonized with MRCoNS (MRSH and/or MRSE) were co-colonized with MRSA. A previous study reported that MRCoNS colonization might be a protective factor against MRSA colonization [6]; nonetheless, other reports have documented nasal co-colonization of MRSA and MRCoNS, indicating that under certain conditions, these species can coexist [7].

Antibiotic profiling showed many MRS isolates exhibited resistance only to penicillin and oxacillin, aligning with a community-associated origin, and resistance to additional antibiotics was observed in individual strains, probably representing sporadic cases. Notably, MRCoNS of this study exhibited distinct resistance (higher resistance rates to tetracycline and sulfamethoxazole-trimethoprim [tigecycline in MRSH]) and broader antibiotic resistance profiles compared to MRSA. This is concerning, as MRCoNS are known to be reservoirs for dissemination of resistance genes and SCC*mec* elements, facilitating the spread of antibiotic resistance [8]. Most studies found MRSE to predominantly harbor SCC*mec* type IV, while MRSH carries SCC*mec* type V [9]. However, in contrast to these trends, most MRSH isolates in this study harbored a combination of SCC*mec* type I and V. Interestingly, an earlier report from Klang Valley, Malaysia, also found the co-occurrence of SCC*mec* type I and V in local MRSH isolates from healthy adults [10], suggesting that these genotypes are possibly circulating within the community, warrant further investigation into MRCoNS transmission within the population.

This study has some limitations, including potential selection and geographical biases due to the restricted sampling area and limited migrant representation. Nonetheless, to the best our knowledge, this is the first MRS community screening among migrant workers in Klang Valley, reporting a nasal MRS carriage rate of 14.7%. Distinct MRS species dominance (MRSH in Indonesian, MRSA in Bangladeshi) and higher carriage associated with crowded settings, suggests potential silent transmission and underscores the importance of continued surveillance.

## Declaration of competing interest

The authors have no competing interests to declare.

## Appendix Supplementary materials

Image, application 1
