# Supplementary material for: Community carriage of methicillin-resistant staphylococci among migrant communities living in Klang Valley, Malaysia
Source: IJID Reg. 2026 Jan 24;18:100849. doi: 10.1016/j.ijregi.2026.100849 (PMC12924174; doi:10.1016/j.ijregi.2026.100849)
Supplement: Supplementary file 1 [file mmc1.docx]

**SUPPLEMENTARY MATERIALS**

**Participant recruitment**

Migrant workers originating from Indonesia, Bangladesh, and Nepal who were living in Klang Valley were recruited through the North-South Initiative (NSI), a Malaysian non-governmental organisation (https://nsinitiative.net/) between December 2023 and May 2024. Potential participants were informed about the study via WhatsApp messages disseminated by their community migrant leaders through NSI liaison. A health seminar was then jointly organised with NSI at the NSI headquarters, where participants first received education on antibiotic use and hygiene practices. Following this, participants were briefed on the study prior to consent taking and sociodemographic data collection. Sample collection was then conducted, where one nasal swab (both anterior nares) was obtained from each participant. Swabs were transported in modified Amies transport medium (Vacutest Kima, Italy) at room temperature and processed within 24 hours. As this study was a pilot-based investigation and involved migration sensitivities (participants were concerned about work permit renewals should they be identified carrying MRS), we targeted a minimum sample size of 30 participants per nationality to provide sufficient representation for preliminary insights into the study research questions [1].

**DNA extraction for PCR amplification**

DNA extractions were performed via lysostaphin digestion. Briefly, overnight cultures were pelleted via centrifugation at 15,000 rpm for 1 minute and the supernatant was discarded. The remaining pellets were resuspended in 180 µL of TE buffer with 2 µL of 2 mg/ml of lysostaphin and incubated for 1 hour or until a clear solution was obtained. The extracted DNA was use for species identification, *mec*A gene detection and SCC*mec* typing via PCR.

**Staphylococcal species identification and methicillin resistance (*mec*A gene) detection**

Species identification was confirmed using PCR with species-specific primers as outlined in Supplementary Table 1. PCR amplification was performed to identify *Staphylococcus spp. , Staphylococcus aureus*, *Staphylococcus epidermidis*, and *Staphylococcus haemolyticus*. To confirm methicillin resistance, the presence of the *mecA* gene was detected through PCR amplification using specific primers targeting the *mecA* gene.

**Supplementary Table 1** List of primers used for PCR amplification

| **Target** | **Primer** | **Nucleotide sequence (5′→3′)** | **Annealing temperature** | **Reference** |
| --- | --- | --- | --- | --- |
| Methicillin resistance | mA1 | TGCTATCCACCCTCAAACAGG | 48°C | [2] |
| (*mecA* gene) | mA2 | AACGTTGTAACCACCCCAAGA |  |  |
| *Staphylococcus spp* | TstaG422 | GGCCGTGTTGAACGTGGTCAAATCA | 55°C | [3] |
| (*tuf* gene) | Tstag765 | TIACCATTTCAGTACCTTCTGGTAA |  |  |
| *S. aureus* | Spa-F | AGCACCAAAAGAGGAAGACAA | 55°C | [4] |
| (*spaA* gene) | Spa-R | GTTTAACGACATGTACTCCGT |  |  |
| *S. epidermidis* | Se705-1 | ATCAAAAAGTTGGCGAACCTTTTCA | 53°C | [3] |
| (Se705 fragments) | Se705-2 | CAAAAGAGCGTGGAGAAAAGTATCA |  |  |
| *S. haemolyticus* | SH1 | GGT CGC TTA GTC GGA ACA AT | 56°C | [5] |
| (*mvaA* gene) | SH2 | CAC GAG CAA TCT CAT CAC CT |  |  |

**Antibiotic susceptibility testing**

Antibiotic susceptibilities testing was determined for all MRS strains included in this study using VITEK® 2 automated system (bioMérieux, France) using AST-GP67 panel card (*Staphylococcus spp*.) according to the Clinical Laboratory Standard Institute (CLSI) guidelines. The panel card includes the following antibiotics: Benzylpenicillin, cefoxitin, oxacillin, ciprofloxacin, erythromycin, chloramphenicol, daptomycin, gentamicin, linezolid, mupirocin, nitrofurantoin, rifampicin, teicoplanin, tetracycline, tigecycline, trimethoprim, vancomycin and clindamycin.

**SCC*mec* typing**

SCC*mec* typing was performed using multiplex PCR as described previously [6] using primers listed in Supplementary Table 2. The PCR was performed in a Bio-Rad T100 Thermal Cycler (Bio-Rad, USA) with one cycle of initial denaturation at 94°C for 4 minutes, 30 cycles of denaturation at 94°C for 30 seconds, annealing for 30 seconds at 55°C, extension at 72°C for 1 minute, followed by one cycle of a final extension at 72°C for 4 minutes. The amplification products were resolved by 1.2% (w/v) agarose gel electrophoresis for 60 minutes at 85V and visualized with Quantum ST4 Gel Documentation System (Vilber Lourmat France).

**Supplementary Table 2** List of primers used for SCC*mec* typing

| **Name** | **Primer sequence (5′→3′)** | **Length** | **Target** | **SCC*mec* type^a^** | | | | |
| --- | --- | --- | --- | --- | --- | --- | --- | --- |
|  |  |  |  | **I** | **II** | **III** | **IV** | **V** |
| β | ATTGCCTTGATAATAGCCYTCT | 937 bp | *ccrA2‐B* |  | X |  | X |  |
| α3 | TAAAGGCATCAATGCACAAACACT |  |  |  |  |  |  |  |
| ccrCF | CGTCTATTACAAGATGTTAAGGATAAT | 518 bp | *ccrC* |  |  | X |  | X |
| ccrCR | CCTTTATAGACTGGATTATTCAAAATAT |  |  |  |  |  |  |  |
| 1272F1 | GCCACTCATAACATATGGAA | 415 bp | IS*1272* | X |  |  | X |  |
| 1272R1 | CATCCGAGTGAAACCCAAA |  |  |  |  |  |  |  |
| 5RmecA | TATACCAAACCCGACAACTAC | 359 bp | *mecA*–IS*431* |  |  |  |  | X |
| 5R431 | CGGCTACAGTGATAACATCC |  |  |  |  |  |  |  |

^a^ “X” denotes positive bands in indicated SCC*mec* types

**References**

[1]  Browne RH. On the use of a pilot sample for sample size determination. Stat Med. 1995 Sep 15;14(17):1933–40.

[2]  Kondo Y, Ito T, Ma XX, Watanabe S, Kreiswirth BN, Etienne J, et al. Combination of multiplex PCRs for staphylococcal cassette chromosome mec type assignment: rapid identification system for mec, ccr, and major differences in junkyard regions. Antimicrob Agents Chemother. 2007 Jan;51(1):264–74.

[3]  Morot-Bizot SC, Talon R, Leroy S. Development of a multiplex PCR for the identification of Staphylococcus genus and four staphylococcal species isolated from food. Journal of Applied Microbiology. 2004;97(5):1087–94.

[4]  Sabat A, Krzyszton-Russjan J, Strzalka W, Filipek R, Kosowska K, Hryniewicz W, et al. New Method for Typing Staphylococcus aureus Strains: Multiple-Locus Variable-Number Tandem Repeat Analysis of Polymorphism and Genetic Relationships of Clinical Isolates. J Clin Microbiol. 2003 Apr;41(4):1801–4.

[5] Schuenck RP, Pereira EM, Iorio NLP, Dos Santos KRN. Multiplex PCR assay to identify methicillin-resistant Staphylococcus haemolyticus. FEMS Immunol Med Microbiol. 2008 Apr;52(3):431–5.

[6] Boye K, Bartels MD, Andersen IS, Møller JA, Westh H. A new multiplex PCR for easy screening of methicillin-resistant Staphylococcus aureus SCCmec types I-V. Clin Microbiol Infect. 2007 Jul;13(7):725–7.
